# Increased Cerebrospinal Fluid Adenosine 5′-Triphosphate Levels in Patients with Guillain–Barré Syndrome and Chronic Inflammatory Demyelinating Polyneuropathy

**DOI:** 10.1155/2024/7229216

**Published:** 2024-06-10

**Authors:** Takamasa Nukui, Hideki Niimi, Tomohiro Hayashi, Nobuhiro Dougu, Mamoru Yamamoto, Ryoko Shibuya, Noriyuki Matsuda, Ryo Tanaka, Hiroaki Hirosawa, Risako Furuta, Taichi Mitsui, Hiroki Maesaka, Syuhei Takasawa, Isao Kitajima, Yuji Nakatsuji

**Affiliations:** ^1^Department of Neurology, Faculty of Medicine, University of Toyama, Toyama, Japan; ^2^Department of Clinical Laboratory and Molecular Pathology, Graduate School of Medicine and Pharmaceutical Science for Research, University of Toyama, Toyama, Japan; ^3^Takaoka City Hospital, Takaoka, Japan

## Abstract

**Background:**

Extracellular adenosine 5′-triphosphate (ATP) acts as a signaling molecule in the peripheral nerves, regulating myelination after nerve injury. The present study examined whether the cerebrospinal fluid (CSF) ATP levels in patients with Guillain–Barré syndrome (GBS) and chronic inflammatory demyelinating polyneuropathy (CIDP) are related to disease severity.

**Methods:**

CSF ATP levels in 13 patients with GBS and 18 patients with CIDP were compared with those in a control group of 16 patients with other neurological diseases (ONDs). In patients with CIDP, CSF ATP levels were compared before and after treatment. The correlations between CSF ATP levels and other factors, including clinical data and CSF protein levels, were also evaluated.

**Results:**

Median CSF ATP levels were significantly higher in patients with GBS and CIDP than in those with ONDs. When patients with CIDP were classified into two groups depending on their responsiveness to immunotherapy, median CSF ATP levels were significantly higher in good responders than in ONDs. CSF ATP levels tended to decrease after treatment in patients with CIDP. In patients with CIDP, there is a negative correlation between CSF ATP and CSF protein levels.

**Conclusions:**

CSF ATP levels were increased in patients with GBS and CIDP. In particular, CSF ATP levels tended to decrease following treatment in patients with CIDP. CSF ATP levels may be useful biomarkers for the diagnosis or monitoring of therapeutic effects in patients with GBS and CIDP.

## 1. Introduction

Guillain–Barré syndrome (GBS) and chronic inflammatory demyelinating polyneuropathy (CIDP) are both immune-mediated demyelination neuropathies. In GBS, leukocytes infiltrate peripheral nerves via an autoimmune mechanism associated with antecedent infection, leading to the rapid progression of limb paralysis and sensory disturbance [1]. CIDP is characterized by slowly progressive limb weakness and sensory disturbances lasting for >2 months. Although the immunological pathogenesis of this disease remains poorly understood, evidence suggests that aberrant cellular and humoral immunity cause peripheral nerve demyelination and axonal injury [2]. Although patients with GBS and CIDP sometimes present with severe symptoms and poor prognosis, these are treatable diseases, for which early diagnosis and treatment are very important. The diagnosis of these diseases is predominantly based on clinical symptoms and electrophysiological examinations; however, blood and cerebrospinal fluid (CSF) biomarkers can also be useful. Several blood and CSF proteins associated with demyelination have been investigated as biomarkers of GBS and CIDP. For example, CSF sphingomyelin levels, which are useful diagnostic biomarkers, are elevated in patients with acquired demyelinating neuropathies [3]. In patients with GBS, serum neurofilament light chain (NfL) levels are correlated with clinical outcomes [4]. Similarly, serum NfL levels were found to be increased in patients with CIDP and were decreased during the remission period [5]. However, there are still no established biomarkers to reflect disease severity or monitor the therapeutic effects in patients with GBS and CIDP.

Extracellular adenosine 5′-triphosphate (ATP) acts as a signaling molecule in the peripheral nervous system by binding to its specific receptor. Under physiological conditions, neurons release ATP, which regulates myelination by activating Schwann cell proliferation via action on purinergic receptors. Under pathological conditions, ATP is released from dying cells and regulates myelination following nerve injury [6]. The relationship between CSF ATP levels and the pathophysiology of several neurological diseases has been investigated. In patients with mitochondrial myopathy, encephalopathy, lactic acidosis, and stroke-like episodes (MELAS), CSF ATP levels have been found to reflect disease severity and treatment efficacy [7]. Other studies have further reported that CSF ATP levels are elevated in patients with neuromyelitis optica spectrum disorder (NMOSD), which induces neuropathic pain [8]. Furthermore, elevated CSF ATP levels have been associated with disease severity in amyotrophic lateral sclerosis [9]. However, the relationship among CSF ATP levels, disease severity, and response to treatment has not been investigated in GBS and CIDP. The present study examined whether CSF ATP levels in patients with GBS and CIDP are related to disease severity or other clinical factors.

## 2. Materials and Methods

### 2.1. Patients and Ethics

Thirteen patients with GBS and eighteen patients with CIDP admitted to the Toyama University Hospital between 2008 and 2022 were enrolled. GBS was diagnosed according to the Brighton criteria [10]. All patients fulfilled the diagnostic certainty level of 1 or 2 and tested positive for antiganglioside antibodies. Patients with CIDP were newly diagnosed according to the European Federation of Neurological Societies/Peripheral Nerve Society (EFNS/PNS) 2010 criteria [11] and had no history of prior immunotherapy. To compare CSF ATP levels before and after treatment in patients with CIDP, initial CSF specimens were sampled at the time of diagnosis, prior to the induction of immunotherapy at the Toyama University Hospital. Posttreatment CSF was collected when the patients were readmitted for the second course of immunotherapy. The median interval between the CSF collections was 5 months. CSF samples from patients with other neurological diseases (ONDs) (*n* = 16), including idiopathic normal-pressure hydrocephalus (iNPH, *n* = 11), familial amyloid polyneuropathy (*n* = 4), and neuropathy with a liability to pressure palsy (*n* = 1), were used as controls. As CSF ATP levels increase due to pleocytosis and blood contamination, CSF samples collected by traumatic lumbar punctures were excluded. This study was conducted in accordance with the Code of Ethics of the World Medical Association (Declaration of Helsinki) for experiments involving humans and was approved by the Ethics Committee of the University of Toyama (approval no. 29–32). Written informed consent was obtained from all the patients.

### 2.2. Measurement of the Extracellular ATP Levels

CSF samples were obtained from lumbar puncture and stored at −80°C until measurement. A highly sensitive and automated ATP measurement device was used to measure extracellular ATP levels [12]. Luciferin-luciferase reagent HS from the Lucifell HS Set (Kikkoman Biochemifa Co., Ltd., Tokyo, Japan) was selected as the luminescent reagent and diluted 10-fold with distilled water. The CSF specimens were diluted 20-fold with distilled water before measurement to prevent the inhibitory effect of high concentrations of chloride ions on the luminescent reagent. Luminescence was measured using a luminometer for 10 s after the addition of 50 *µ*L of luminescent reagent to 10 *µ*L of the diluted specimen. Extracellular ATP levels were quantified as the average relative light intensity (counts/s). Measurements were performed in triplicate for each sample. A calibration curve for the luminescence and ATP concentrations were obtained using a 10-fold dilution series of a standard ATP solution adjusted with a saline solution diluted 10-fold with distilled water. ATP concentrations were further calculated from the luminescence values obtained from the calibration curve [7]. To confirm the precision and detection limit of the assay, we measured ATP standard solutions prepared using commercial ATP and CSF collected from patients using iNPH as the diluent. These results indicated that the measurement error reached up to 2.4%, while the ATP quantification limit in the CSF was at least 2.0 × 10^−11^ mol/L.

### 2.3. Statistical Analysis

Statistical tests were performed using the JMP^Ⓡ^ Pro software 2019 ver 15.0 (SAS Institute Inc., Cary, NC, USA). The Wilcoxon test with Bonferroni correction was used for comparison between groups. Fisher's exact test was used to compare the differences in frequency distribution according to sex. Spearman's test was used to examine the correlations between variables. The Wilcoxon signed-rank test was used to compare the paired data. Results were considered significant when the *p* values were <0.05.

## 3. Results

### 3.1. Clinical Characteristics and CSF Profiles of Patients with GBS and CIDP

**Figure 1:**
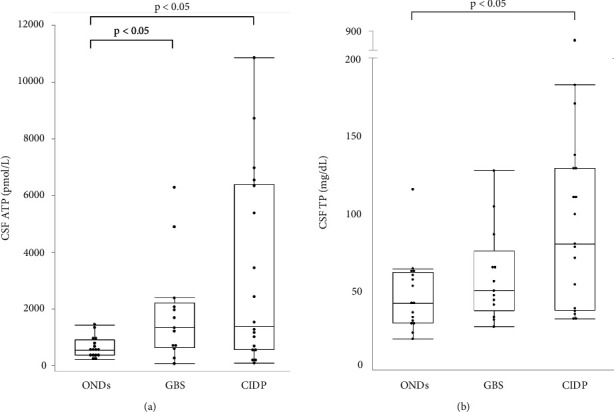
(a) The boxplot of CSF ATP levels in GBS and CIDP patients. The median (interquartile range, IQR) CSF ATP levels was 549 (368–910) vs. 1330 (637−2,205) vs. 1,382 (555−6,390) pmol/L in ONDs, GBS, and CIDP, respectively. Steel test was used for comparing the groups. Values of *p* < 0.05 were statistically significant. (b) The boxplot of CSF TP levels in GBS and CIDP patients. The median (IQR) CSF TP levels were 43 (30.25–62.5) vs. 51 (38–76.5) vs. 90.5 (38.75–132) mg/dL in ONDs, GBS, and CIDP, respectively. Wilcoxon test with Bonferroni correction was used for comparing the groups. Values of *p* < 0.05 were statistically significant.

The median ages of patients with GBS and CIDP were 49 and 62.5 years, respectively, both of which were lower than that of patients with ONDs. Patients with GBS were classified according to Ho's electrodiagnostic criteria [13]; four cases (30.8%) were demyelinating, four (30.8%) were axonal, and five (38.4%) were unclassifiable because of a lack of sufficient electrophysiological tests. Eleven (61.1%) patients with CIDP had typical CIDP, while seven (38.9%) had atypical CIDP. The median Medical Research Council (MRC) sum score in patients with CIDP was significantly higher than that in patients with GBS (54.5 vs. 44, respectively, *p* < 0.05). The median Hughes grade of patients with GBS was 3 (interquartile range (IQR), 2.5–4). The median modified Rankin Scale (mRS) and Inflammatory Neuropathy Cause and Treatment Disability (INCAT) scores of the patients with CIDP were 3 (IQR, 2–3.25) and 3.5 (IQR, 2.75–5), respectively (Table 1). The median CSF ATP levels in patients with GBS and CIDP were 1,330 (IQR, 637−2,205) and 1,382 (IQR, 555−6,390) pmol/L, respectively, both of which were significantly higher than those in patients with ONDs (Figure 1(a)). There were no significant differences in CSF ATP levels according to the disease type in either GBS or CIDP (data not shown). The median CSF total protein (TP) level was significantly higher in patients with CIDP than in those with ONDs (90.5 vs. 43 mg/dL, *p* < 0.05) (Figure 1(b)). The cerebrospinal fluid albumin/serum albumin (Qalb) ratio, which reflects impairment of the blood-brain barrier, was significantly higher in patients with CIDP than in those with GBS (14.34 vs. 8.25, *p* < 0.05) (Table 2).

### 3.2. Correlation between CSF ATP Levels and Other Variables in Patients with GBS and CIDP

**Figure 2:**
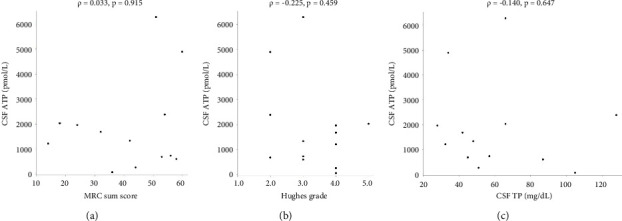
Correlations between the CSF ATP levels and MRC sum score (a), Hughes grade (b), and CSF TP (c) in patients with GBS. Spearman's rank correlation was used for analysis.

**Figure 3:**
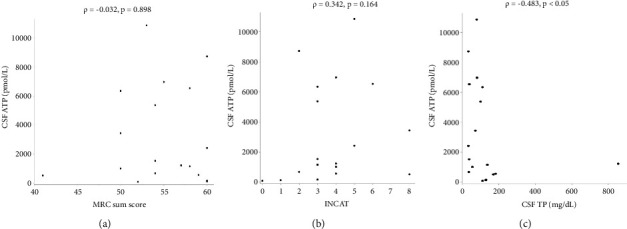
Correlations between the CSF ATP levels and MRC sum score (a), INCAT (b), and CSF TP (c) in patients with CIDP. Spearman's rank correlation was used for analysis.

There were no statistically significant correlations between CSF ATP levels and the MRC sum score, Hughes grade, or CSF protein levels in patients with GBS (Figures 2(a)–2(c)). Among patients with CIDP, a negative correlation between CSF ATP and CSF protein levels was found although there was no correlation between CSF ATP levels and the MRC sum score, INCAT (Figures 3(a)–3(c)). Other factors, such as age, CSF cells, IgG index, Qalb, and electrophysiological examination results, including nerve conduction velocity, distal latency, and compound muscle action potential of the median or tibial nerve, were not significantly correlated with CSF ATP levels in patients with GBS and CIDP (data not shown).

### 3.3. Relationship between CSF ATP Levels and Immunotherapy in Patients with CIDP

**Figure 4:**
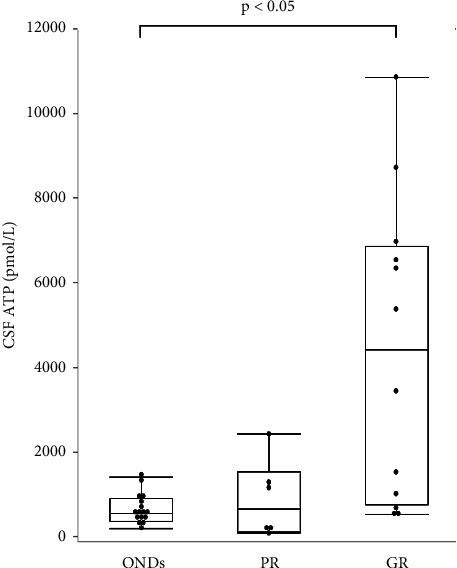
The boxplot of CSF ATP levels in ONDs and CIDP patients (poor responders, PR *n* = 6; good responders, GR *n* = 12). The median (IQR) CSF ATP levels were 549 (368–910) vs. 663 (118−1,533) vs. 4,408 (767−6,863) in ONDs, NR, and GR, respectively. Steel test was used for comparing the groups. Values of *p* < 0.05 were statistically significant. GR; clinical improvement documented by the change of at least one point in the clinical scales (INCAT, ONLS, and mRS) by immunotherapy.

**Figure 5:**
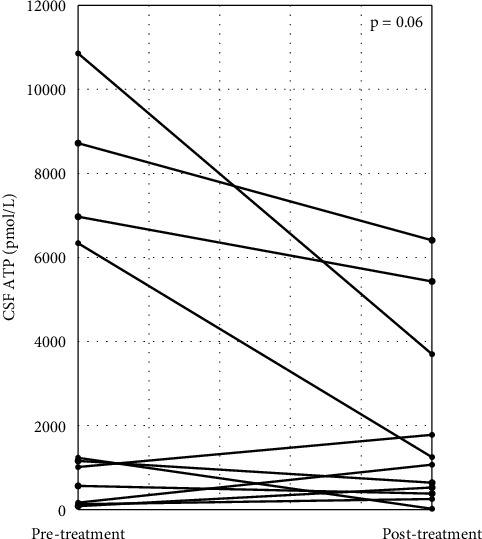
Changes in CSF ATP levels and CSF TP levels in each individual patient with CIDP (*n* = 11) at different disease phases of pretreatment and posttreatment.

Patients with CIDP were classified into two groups, depending on their responsiveness to immunotherapy. Poor responders (PRs) included patients whose clinical scales (INCAT, ONLS, and mRS) did not change after immunotherapy, while good responders (GRs) showed clinical improvement, documented by a change of at least one point in the clinical scale by immunotherapy. In the GR group, seven patients (58.3%) had typical CIDP, while in the PR group, four patients (66.7%) had typical CIDP, with no significant differences in disease type between the groups. The median CSF ATP levels were significantly higher in the GR group than in the ONDs group (549 (IQR, 368–910) vs. 4408 (IQR, 767–6863) pmol/L, *p* < 0.05) (Figure 4). CSF ATP levels before and after treatment were compared in 11 patients with CIDP for whom CSF samples were available. The CSF ATP levels tended to decrease in each patient with CIDP after treatment, although the difference did not reach statistical significance (Figure 5).

## 4. Discussion

This study demonstrated that CSF ATP levels were significantly higher in patients with GBS and CIDP than in controls, while CSF ATP levels were higher in patients with CIDP who responded well to immunotherapy and tended to decrease in each patient after treatment. To the best of our knowledge, this is the first study to evaluate the CSF ATP levels in patients with GBS and CIDP.

Previous studies reported that extracellular ATP is involved in the regulation of myelination after nerve injury. For example, extracellular ATP levels are increased in *in vitro* Wallerian degeneration models, where they inhibit myelin sheath degradation [14]. Extracellular ATP secreted from degenerating neurons elicited an increase in cytosolic Ca^2+^ levels in Schwann cells and activated Schwann cell regeneration in an *in vitro* model of the Miller Fisher syndrome, a subtype of GBS [15]. In contrast, *in vitro* experiments have shown that a high concentration of extracellular ATP activates microglia and astrocytes by binding to specific receptors and inducing neurotoxic inflammation [16]. Therefore, extracellular ATP receptor antagonists have shown to have potential as novel therapies for reducing microglial activity and neuroinflammation in several neurological diseases including GBS [17], epilepsy [18], Parkinson's disease [19], and Alzheimer's disease [20]. These findings suggest that increased extracellular ATP acts as a damage-associated molecule and promotes peripheral nerve regeneration or causes peripheral nerve damage in some demyelinating neuropathies such as GBS and CIDP. Although no previous studies have measured the CSF ATP levels in patients with inflammatory neuropathy, Ishikura et al. measured CSF ATP levels in patients with NMOSD, an inflammatory spinal cord disease [8]. In that report, the mean CSF ATP level in patients with NMOSD was approximately 8,000 pmol/L, which was higher than that in our study (mean 1,853 pmol/L in patients with GBS and 3,209 pmol/L in patients with CIDP). This suggests that CSF ATP levels may vary depending on the degree and location of inflammation.

In the present study, we identified a negative correlation between CSF ATP and CSF protein levels in patients with CIDP although there was no correlation between CSF ATP levels and the MRC sum score or clinical scores, such as Hughes grade and INCAT, in patients with GBS and CIDP. Albuminocytological dissociation provided evidence supporting the diagnosis of CIDP [21]. As albuminocytological dissociation indicates a disruption of the blood-nerve barrier due to nerve inflammation [22], high CSF protein levels may suggest a high degree of nerve inflammation and nerve injury. Furthermore, because high CSF ATP levels likely occur as a result of ATP secretion from injured neurons, a positive correlation is expected between CSF ATP levels and CSF protein levels. Although the reason for this negative correlation is unclear, the small number of patients may be the main reason.

In addition, CSF ATP levels tended to decrease after immunotherapy in patients with CIDP. This indicates that CSF ATP levels may be a therapeutic biomarker in patients with CIDP. To date, no therapeutic biomarkers have yet been established for immunotherapy in patients with CIDP. NfL is an abundant cytoskeletal component of axons and a useful biomarker for peripheral nervous system diseases [23]. Kapoor et al. further reported that the plasma NfL concentration was higher in patients with CIDP than in healthy controls and was significantly reduced after IVIg therapy [24]. As they also showed that plasma NfL concentration was significantly higher in unstable patients with CIDP than in stable patients during IVIg treatment, plasma NfL was considered to be a useful biomarker reflecting axonal injury in patients with CIDP. Sphingomyelin (SM), a myelin-rich lipid, is a component of myelin sheaths. In the CSF of patients with acquired demyelinating neuropathies, several pathological products are increased owing to their release from nerve roots or flow from peripheral blood, which is attributed to the disruption of the blood-nerve barrier that reflects damage to peripheral nerves [25]. In patients with GBS and CIDP, CSF sphingomyelin levels were found to be increased compared to those in patients with nondemyelinating neurological diseases and were further higher in patients with active CIDP than in those with stable disease [26]. These data suggest that CSF sphingomyelin acts as a diagnostic and severity biomarker of GBS and CIDP. Compared with NfL and sphingomyelin, both of which indicate peripheral nerve injury, CSF ATP levels are elevated upon remyelination and may be a marker of remyelination. CSF ATP levels may be more useful than NfL and sphingomyelin levels in predicting therapeutic efficacy in patients with CIDP.

This study had some limitations. First, the sample size was relatively small, and the relationships between CSF ATP levels and clinical scores, disease severity, and electrophysiological examinations in patients with GBS and CIDP could not be elucidated. Furthermore, type 2 errors were likely to occur because of small sample sizes. Second, there were no data on the correlation between CSF ATP levels and other biomarkers such as CSF NfL. If elevated CSF ATP levels are associated with peripheral nerve regeneration, then a negative correlation may be observed between CSF ATP and NfL levels in patients with GBS and CIDP. Third, multivariate correlation analysis was not performed because of the small number of cases and due to the potential for unknown confounding factors that affect CSF ATP concentration, such as age and underlying disease. Fourth, the change in CSF ATP levels in patients with CIDP was only measured between two time points, before and after treatment, and there were no data on the long-term follow-up. As this was a pilot exploratory study, continuous sampling over longer treatment periods in larger cohorts is required to demonstrate the utility of CSF ATP levels as a therapeutic biomarker in patients with CIDP, similar to other biomarkers such as NfL and sphingomyelin.

## 5. Conclusion

Overall, the present study showed that CSF ATP levels were increased in patients with GBS and CIDP. CSF ATP levels may be useful biomarkers for the diagnosis or monitoring of therapeutic effects in patients with GBS and CIDP.

## Figures and Tables

**Table 1:** Demographics of the ONDs and patients with GBS CIDP.

	ONDs (*n* = 16)	GBS (*n* = 13)	CIDP (*n* = 18)	*P* value
Age (year)	72 (69–74.75)	49 (36.5–65)	62.5 (50.25–70.25)	<0.01^*∗*^
Male (%)	11 (68.8)	6 (46.2)	9 (50.0)	0.46
MRC sum score		44 (28–55)	54.5 (51.5–59.25)	<0.05^*∗*^
Hughes grade		3 (2.5–4)		
mRS			3 (2–3.25)	
INCAT (upper + lower)			3.5 (2.75–5)	

MRC sum score: Medical Research Council sum score; mRS: modified Rankin Scale; INCAT score: Inflammatory Neuropathy Cause and Treatment Disability Score. Data are presented as the median (interquartile range) or number (%). ^*∗*^Indicates *p* < 0.05 (Wilcoxon test with Bonferroni correction), ONDs: other neurological diseases included iNPH (idiopathic normal pressure hydrocephalus), FAP (familial amyloid polyneuropathy), and HNPP (hereditary neuropathy with liability to pressure palsy). GBS: Guillain–Barré syndrome and CIDP; chronic inflammatory demyelinating polyneuropathy.

**Table 2:** CSF data of the ONDs and the patients with GBS and CIDP.

	ONDs (*n* = 16)	GBS (*n* = 13)	CIDP (*n* = 18)	*P* value
CSF ATP (pmol/L)	549 (368–910)	1330 (637–2205)	1382 (555–6390)	<0.05^*∗*^
CSF cells (/*µ*L)	0.5 (0–1)	1 (0–1.5)	1 (0–2)	0.25
CSF TP (mg/dL)	43 (30.25–62.5)	51 (38–76.5)	90.5 (38.75–132)	<0.05^*∗*^
IgG index		0.535 (0.465–0.5875)	0.55 (0.485–0.605)	0.91
Q alb × 10^3^		8.25 (6.38–10.88)	14.34 (7.045–21.5)	<0.05^*∗*^

The GBS group included patients whose CSF samples were collected within four weeks. The CIDP group included patients whose CSF samples were collected prior to immunotherapy. Data are presented as the median (interquartile range). ^*∗*^*p* < 0.05 (Wilcoxon test with Bonferroni correction).

## Data Availability

The datasets used and analyzed in this study are available from the corresponding author upon reasonable request.
